# 606. Clinical Utility of Fungal Blood Cultures for the Diagnosis of Candidemia

**DOI:** 10.1093/ofid/ofad500.672

**Published:** 2023-11-27

**Authors:** Rishika Bheem, Benjamin Hanisch, Benjamin M Liu, Joseph M Campos, Emily Ansusinha, Rana F Hamdy

**Affiliations:** George Washington University School of Medicine and Health Sciences, Washington, District of Columbia; Children’s National Hospital, Washington, DC, USA, Washington, District of Columbia; Children's National Hospital /George Washington University, Washington, District of Columbia; Children's National Hospital, Washington, District of Columbia; Children's National Hospital, Washington, District of Columbia; Childrens National Hospital, Washington, District of Columbia

## Abstract

**Background:**

Candidemia is the most common invasive fungal infection in hospitalized children, and the third most common cause of pediatric nosocomial bloodstream infections. The gold standard for diagnosis of *Candida* bloodstream infections is with blood cultures, and clinicians can order aerobic or fungal blood cultures when suspecting fungemia. The objective of this study is to investigate the additional utility of fungal blood cultures (in addition to aerobic blood cultures) for detecting *Candid*a bloodstream infections.

**Methods:**

This retrospective cohort study analyzed all patients < 21 years of age, admitted to Children’s National Hospital between January 1, 2010, and December 31, 2020, and had a positive fungal blood culture (using BHI-CG plates) for *Candida* species on day 1 of fungemia (Table 1). Demographic data, *Candida* species, the concomitant positive aerobic blood cultures (using the bioMérieux BACT/ALERT blood culture system) were collected from the electronic health records through structured chart review. The time to detection of positive blood cultures was calculated for the fungal and aerobic cultures drawn on day 1 of fungemia. P-value < 0.05 was considered statistically significant.

**Table 1:**
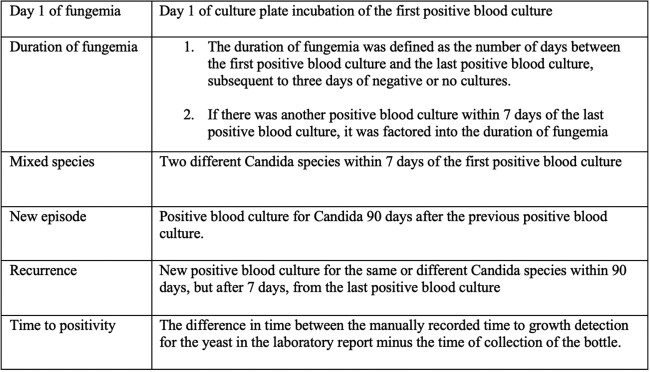
Definitions

**Results:**

Thirty-one episodes of *Candida* fungemia (29 patients) were included in this study, of which 28 episodes also had an aerobic blood culture drawn on day 1 of fungemia that was positive for *Candida* species. All 31 episodes had an aerobic blood culture positive for *Candida* species within the duration of fungemia (Figure 1). The mean age of patients in the cohort was 5.7 ± 6.0 years with mean duration of admission of 67.1 days ± 79.6. *C. parapsilosis* was the most frequently identified *Candida* species (14), followed by *C. albicans* (10), *C. lusitaniae (Clavispora lusitaniae)* (2), *C. tropicalis* (1), *C. krusei* (*Pichia kudriavzevii*) (1) (Table 2). The mean time to positivity for aerobic blood cultures and fungal blood cultures were 26.4 ± 11.7 hours and 76.2 ± 29.6 hours, respectively (p < 0.001).

**Figure 1:**
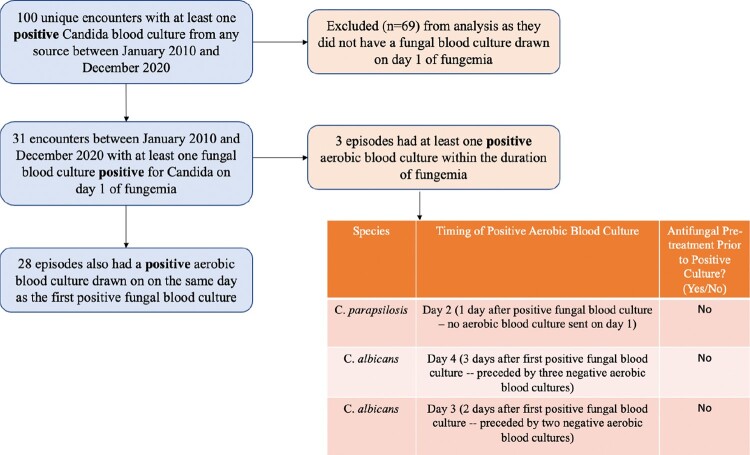
Patient Population and Analysis Flowchart

**Table 2:**
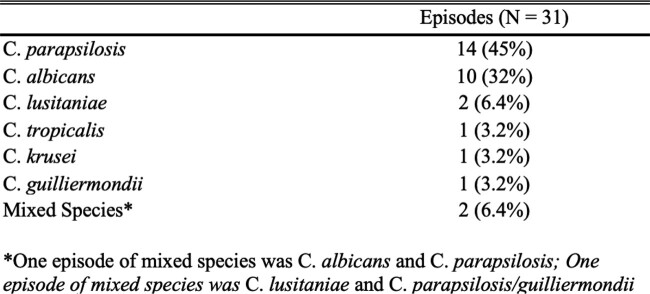
Episodes of Candida by Species

**Conclusion:**

All episodes of *Candida* species that grew on fungal cultures also had at least one aerobic culture positive for *Candida* species within the duration of fungemia. Aerobic blood cultures had a statistically significant shorter time to positivity for *Candida* than fungal blood cultures.

**Disclosures:**

**Benjamin Hanisch, MD**, American Society of Transplantation: Board Member|Astellas Pharma Global Development, Inc.: Support for the present publication|NIH: Site PI for International Pediatric Fungal Network- funds to institution **Joseph M. Campos, PHD**, Accelerate Diagnostics: Honoraria|GenMark Diagnosticd: Honoraria

